# Growth and reproduction of laboratory-reared neanurid Collembola using a novel slime mould diet

**DOI:** 10.1038/srep11957

**Published:** 2015-07-08

**Authors:** Jessica L. Hoskins, Charlene Janion-Scheepers, Steven L. Chown, Grant A. Duffy

**Affiliations:** 1 School of Biological Sciences, Monash University, Clayton, Victoria 3800, Australia

## Abstract

Although significant progress has been made using insect taxa as model organisms, non-tracheated terrestrial arthropods, such as Collembola, are underrepresented as model species. This underrepresentation reflects the difficulty in maintaining populations of specialist Collembola species in the laboratory. Until now, no species from the family Neanuridae have been successfully reared. Here we use controlled growth experiments to provide explicit evidence that the species *Neanura muscorum* can be raised under laboratory conditions when its diet is supplemented with slime mould. Significant gains in growth were observed in Collembola given slime mould rather than a standard diet of algae-covered bark. These benefits are further highlighted by the reproductive success of the experimental group and persistence of laboratory breeding stocks of this species and others in the family. The necessity for slime mould in the diet is attributed to the ‘suctorial’ mouthpart morphology characteristic of the Neanuridae. Maintaining laboratory populations of neanurid Collembola species will facilitate their use as model organisms, paving the way for studies that will broaden the current understanding of the environmental physiology of arthropods.

Much progress in understanding the evolution of environmental tolerances has been made by integrating the outcomes of work on model organisms with investigations of non-model taxa. Work on *Drosophila*, which includes the model *D. melanogaster*, provides a compelling example^1^,^2^. However, to determine how general the findings are from such studies, investigations across a range of other taxa are required. Significant progress has been made in understanding the diversity of environmental responses in other insect taxa^3^,^4^ and non-arthropod groups^5^,^6^,^7^, but for other terrestrial arthropods much remains unknown. This is especially true for non-tracheated arthropods, such as springtails and spiders, and for those that occupy important, but oft-underexplored habitats, such as soil and leaf litter.

The establishment of experimentally tractable populations, comparable to those of *Drosophila*, of novel taxa that occupy understudied habitats will help to address the challenges posed in generalising the findings of model organism studies. The broad geographic distribution of springtails^8^ (Collembola; Arthropoda: Hexapoda), combined with their high abundances (up to 10^5^ m^−2^ in temperate regions^9^) and diversity^10^,^11^, make them ideal organisms to fill this role. At least three Collembola species have been widely established as model organisms (*Folsomia candida*, *Hypogastrura tullbergi,* and *Orchesella cincta*^12^,^13^,^14^,^15^,^16^), with a range of work also being done on field populations of these species^17^,^18^,^19^. A growing body of work likewise concerns the environmental tolerances of a range of springtail species, especially from temperate and polar environments^20^,^21^,^22^,^23^. Nonetheless, much remains to be known about this diverse group, particularly in the context of responses of these soil dwellers to rapidly changing environments^24^.

Successfully maintaining laboratory cultures requires knowledge of how to maintain healthy, disease-free laboratory colonies, and to avoid problems such as inbreeding and laboratory adaptation^25^,^26^. For some springtail species, including the three laboratory models, this knowledge is available given their long history of culture^14^,^27^. However, these species possess the more-typical ‘mandibulate’ mouthpart morphology^8^,^28^ associated with a generalist diet that may vary depending on resource availability^29^,^30^. In contrast, little is known of Collembola species that deviate from the typical ‘mandibulate’ mouthpart morphology, such as those of the family Neanuridae.

Neanuridae, with *c*. 1417 species in 161 genera^31^, comprises about 20% of Collembola species^11^. Unlike their more generalist counterparts, the Neanuridae have ‘suctorial’ mouthparts^28^,^32^,^33^, and have proven difficult to culture in the laboratory. Neanurids apparently incorporate slime moulds into their diet^34^,^35^,^36^,^37^, but whether slime moulds are their primary resource, ensuring growth and survival, or are incidental to their diet remains uncertain. Other arthropod taxa, such as the aptly named slime mould beetles (e.g. *Agathidium* spp.; Coleoptera, Leiodidae), rely almost exclusively on slime mould as a nutrient resource^38^. The rearing of any neanurid Collembola species on a diet of slime mould has not been reported and attempts to keep neanurids in the laboratory on generalist diets have proven unsuccessful, with no feeding or reproductive behaviour observed^36^. In consequence, a diverse family of Collembola, which offers the possibility of additional insights into the evolution and life history of this important class of soil organisms, has to date largely been unavailable for laboratory investigation. Here we directly examine the extent to which Neanuridae can survive, grow, and reproduce on slime moulds using *Neanura muscorum* (Templeton, 1835) as a representative organism with a view to extend this technique to rear other neanurid species. Specifically we establish a broadly applicable rearing method for this family, so improving its tractability as a source of model organisms to broaden current understanding of the environmental tolerances of soil invertebrates.

## Methods

Leaf litter was collected in December 2013 from the Monash University, Clayton campus (Victoria, Australia; 37.9119 °S, 145.1317 °E). Invertebrates were extracted from litter using Berlese-Tullgren funnels^39^. *Neanura muscorum* individuals (n = 31) were identified using morphological characteristics and placed into closed 70 ml containers with a plaster of Paris and charcoal (9:1) substratum. This substratum was regularly saturated with reverse osmosis treated water to maintain humidity^40^. Containers were kept in temperature-controlled rooms set at 15 °C (mean/sd: 14.64 ± 0.61 °C; verified with iButton Hygrochron® temperature/humidity loggers, Maxim Integrated, San Jose, USA) on a 12:12 hour light:dark photoperiod.

**Figure 1:**
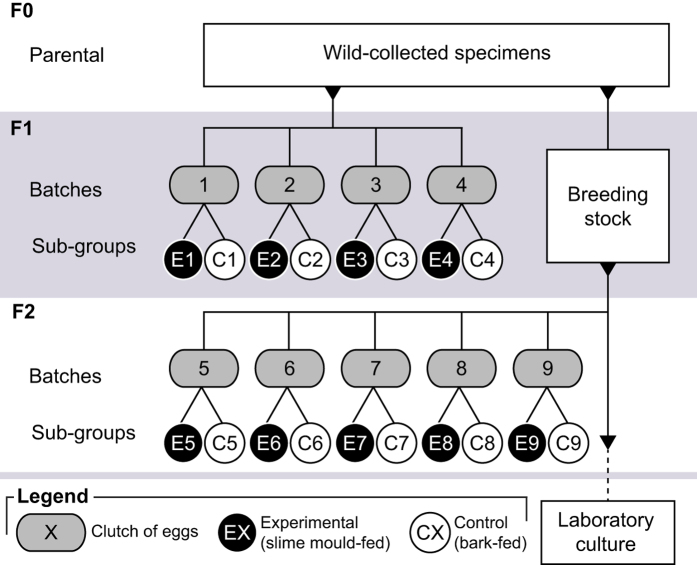
Diagrammatic representation of the experimental design used in this study.

Parental lines (F0) were provided with a combined diet of slime mould (*Physarum polycephalum*; cultured on 1.5% agarose media at 25 °C on a diet of oats) and algae-covered plane tree (*Platanus* sp.) bark *ad libitum*. The latter is a standard diet used in rearing other Collembola species, which enables individuals to select amongst a range of algae, cyanobacteria, and fungi to reach their optimal nutrient target^12^,^13^,^27^. Collembola cultures were checked daily and eggs were removed to new containers and incubated in the temperature-controlled room until hatching. Four batches of first-generation (F1) eggs were used for experiments (Fig. 1). Subsequent clutches were combined and raised as a F1 breeding population under identical conditions to the parental line. Five batches of eggs laid by the F1 breeding population were then used to create F2 populations for a repetition of the experiments undertaken with the F1 generation (Fig. 1). *Neanura muscorum* F2 individuals that were not used for experimentation were pooled and kept as laboratory stocks. These stocks continue to be maintained on a combined slime mould and algae-covered plane tree bark diet.

### Experimental design

On hatching, individuals from each F1 and F2 batch were randomly assigned to either a control or an experimental group and raised in group and batch-specific containers. The control group were exclusively given algae-covered plane tree bark and the experimental group were given only slime mould (*P. polycephalum*). Individuals sharing a container, hereafter referred to as a sub-group (Fig. 1), were of a similar age (±2 days old). In total, four F1 batches and five F2 batches were divided into nine control and nine experimental sub-groups of *N. muscorum* in a pairwise design. Owing to the number of newly hatched individuals available at the founding stage, sub-group size was variable amongst batches (6–40 individuals, median 12.5). However, the pairwise design of this study meant the number of individuals in paired control and experimental sub-groups (e.g. E1 and C1, E2 and C2; Fig. 1) was approximately equal (±1 individual). Food was supplied *ad libitum* and containers were checked every second day to replenish food and water and check for eggs.

**Figure 2:**
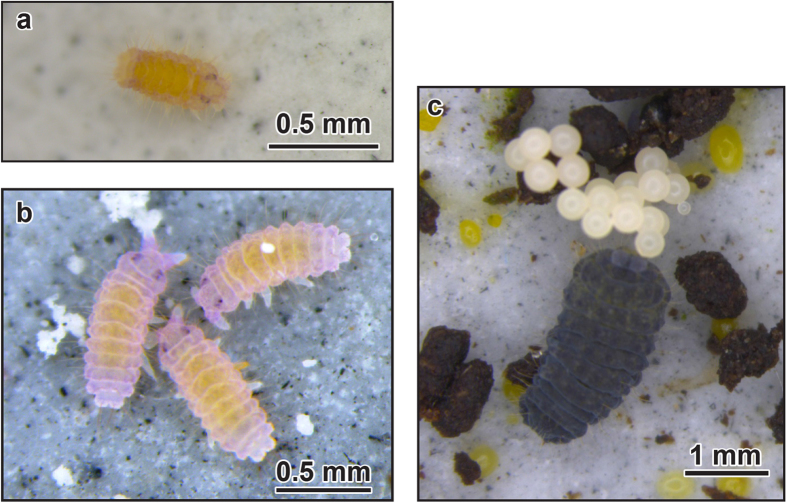
Photographs representing various Neanura muscorum growth-stages. *Neanura muscorum* hatchling (**a** <1 day old), juvenile *N. muscorum* before first moult (**b** <5 days old), and adult *N. muscorum* laying eggs (**c**).

Multiple photographs of each sub-group (e.g. Fig. 2) were taken on a weekly basis using a calibrated microscope (M205C, Leica Microsystems, Wetzlar, Germany). Measurements of total body length (anterior of the head segment to the posterior of the ultimate abdominal segment) were made from these photographs using Leica Application Suite Software (Leica Microsystems). We aimed to photograph and measure at least five individuals from each sub-group. For large sub-groups, one photograph often captured more than five individuals so all specimens within the photograph were measured. Due to mortality, some of the smaller sub-groups contained fewer than five individuals toward the end of the study, in which case all individuals were photographed and measured. Measurements continued on a weekly basis until either the control or experimental sub-group was determined to have reached sexual maturity. The date of first egg-laying was noted for all sub-groups and all eggs were counted and removed to an incubator to determine their viability.

### Statistical Analyses

Second order robust least-squares regression models^41^ were fitted to body-length measurements taken at regular time intervals to produce four separate growth curves for control and experimental F1 and F2 sub-groups. Robust statistics were used to account for data heteroscedasticity. Second order models were chosen to provide the best fit to sub-group growth curves, particularly to capture the expected asymptote in the curves as maturity is reached^12^,^13^. Robust F-tests were used to compare between fitted models and assess the effect of both treatment and generation. Cohen’s d was calculated as a measure of effect size. The effect of treatment as a factor was tested using within-generation comparisons, while generation effects were tested using within-treatment comparisons. Bonferroni correction was applied for all significance testing. All analyses were performed in R statistical software^42^.

## Results

**Figure 3:**
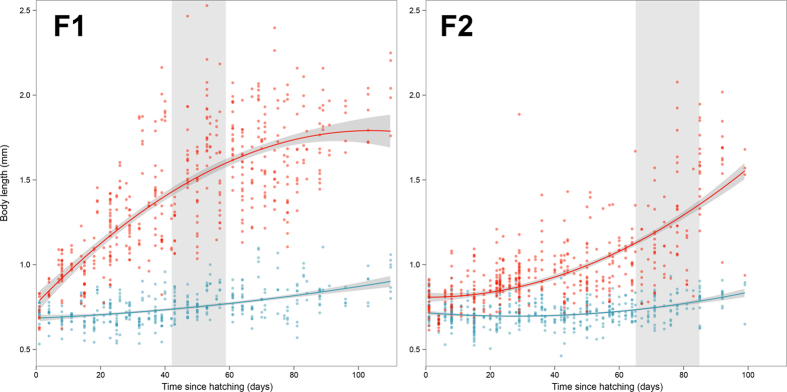
Body length change over time for two generations (F1, F2) of *Neanura muscorum*. Red points represent the experimental group provided with slime mould diet, blue points represent the control group on algae-covered plane tree bark. Fitted models shown with 95% confidence intervals (F1: experimental, y = 1.4205 + 6.1211 x − 1.5328 x^2^, weighted-R^2^ = 0.6906; control, y = 0.7486 + 0.9638 x + 0.1662 x^2^, wR^2^ = 0.6315. F2: experimental, y = 0.9771 + 3.8062 x + 1.0992 x^2^, wR^2^ = 0.6976; control, y = 0.7181 + 0.4565 x + 0.3624 x^2^, wR^2^ = 0.7575. Shaded area represents respective F1 or F2 sub-group age range at first egg laying event.

Only sub-groups of *N. muscorum* given the *P. polycephalum* slime mould diet reached sexual maturity and laid eggs. Six out of nine of these sub-groups laid eggs while the three remaining sub-groups, despite appearing to be of comparable maturity, did not. This may be attributed to the relatively small size of these sub-groups, which was dictated by the number of eggs available at the time of founding. F1 experimental sub-groups laid eggs at a younger age than their F2 counterparts (mean/sd age at first egg laying: 52.5 ± 5.20 and 75 ± 14.14 days respectively; Fig. 3), but individuals in F2 sub-groups were smaller than those in F1 sub-groups when viable eggs were laid.

At the conclusion of the experiment, sub-groups ranged from 78 to 110 days old. The mean body length of individuals from the experimental (slime mould-fed) F1 sub-groups was 1.66 mm (sd ± 0.29 mm), while the control (bark-fed) group had a mean body size of 0.83 mm (sd ± 0.11 mm) at the end of the experimental period. This contrast in final body size of experimental and control sub-groups was mirrored in the F2 generation (1.18 ± 0.27 mm and 0.796 ± 0.09 mm, respectively). Fitted models demonstrated differences in growth rate between treatment groups and generations (Fig. 3). Both the F1 and F2 sub-groups feeding on slime mould showed significantly faster growth rates than their control counterparts (interaction between time since hatching and treatment; F1, F_3, 854_ = 793.62, p < 0.0001, Cohen’s d = 1.9372; F2, F_3, 904_ = 363.51, p < 0.0001, d = 1.2657). Generation effects were also observed with F1 individuals growing faster than those in the F2 sub-groups (interaction between time since hatching and generation; slime, F_3, 941_ = 628.33, p < 0.0001, d = 1.631; bark, F_3, 817_ = 75.44, p < 0.0001, d = 0.6082). As of February 2015 *N. muscorum* laboratory stocks had produced fourth generation (F4) individuals.

## Discussion

The importance of slime moulds in the diet of the Neanuridae has been suggested previously, but clear evidence for them as a primary food source has been lacking. Owing to the similarities in the habitats occupied by neanurid Collembola and slime moulds, both are frequently found in in damp litter, soil, or logs^8^,^43^, it is perhaps unsurprising that Neanuridae can utilise slime mould as a nutritional resource. Anecdotal observations^34^,^35^,^36^,^37^ and stable isotope studies^44^,^45^ have lent support to the idea that they might be important, but here we present explicit evidence that at least one species of the family, *Neanura muscorum*, is able grow and reproduce when given a diet of slime mould. When given slime mould, significant gains in growth rate over bark-fed counterparts were observed. The benefits of a slime mould diet are further highlighted by the reproductive success of the experimental group. Six out of nine experimental sub-groups given slime mould produced viable eggs and healthy, reproductively active sub-groups of *N. muscorum* continue to persist in the laboratory on a diet including a slime mould. In contrast, growth is inhibited and no reproduction occurs when the slime mould resource is absent.

Based on the shared mouthpart morphology across the Neanuridae it is likely that the dependence on slime mould holds true for other members of the family. Previous studies on mouthpart morphology^32^,^33^ and feeding behaviour^34^,^36^,^37^ of other members of this family further suggest that this is the case. Members of the Neanuridae have ‘suctorial’ mouthparts (*sensu* Macnamara^28^) and, unlike the majority of Collembola families, lack a molar plate, which is necessary to process hard food items^46^. The molar plate enables non-neanurid Collembola to subsist on a generalist diet^47^,^48^, and hence be reared on standard algae-covered bark and yeast diets^12^,^13^,^37^. By contrast, Neanuridae mouthparts form a tube-like structure^49^,^50^ that is better suited for piercing and sucking^8^. Neanurids also lack the necessary enzymes to digest fungal cell walls^47^ and stable-nitrogen isotope analyses indicate that Neanuridae species have higher protein content in their diets than other springtail taxa^44^,^45^. Therefore, neanurid springtails require protein-rich fluid-based diets or small particles in suspension^10^,^47^,^51^. Slime moulds clearly fit this description.

As evidenced here, *P. polycephalum* is a viable food source for lab-rearing *N. muscorum*. *Neanura muscorum* is one of the most common and widespread Collembola in Europe and is invasive in many parts of the world^52^, including Australia. It is also the only member of the subfamily Neanurinae that is known to be parthenogenetic^53^,^54^. It can therefore be argued that *N. muscorum* is easier to keep and breed than other species of Neanuridae owing to the broad environmental tolerance associated with being invasive, and the reproductive flexibility afforded by parthenogenesis^54^. However, when used to supplement the standard algae-covered bark diet, slime mould has also been used to rear six other species across different tribes in the Neanuridae (Table 1; Supplementary Information online). The successful rearing of other, indigenous and non-parthenogenetic, Neanuridae species demonstrates that *N. muscorum* represents the rule rather than the exception.

The intergenerational differences in growth-rate and onset of reproduction found for *N. muscorum* may be due to differences in maternal and grandmaternal investment, although other factors may also be important. F1 individuals that were fed the slime mould *P. polycephalum* grew significantly faster than their F2 counterparts and reproduced at a much younger age. However, when F2 individuals ultimately reached reproductive maturity they were substantially smaller than reproductively active F1 individuals. Although less pronounced, differences in growth rate were mirrored in the control, bark-fed, group. *Folsomia candida* exhibits remarkable flexibility in its reproductive investment in response to food supply and crowding^55^, and food availability influences age at maturity and reproductive output through maternal and grandmaternal effects^56^. Comparable transgenerational effects arising from nutrient variability have been found to span at least two generations in *Drosophila melanogaster*^57^,^58^. The parents of F1 and F2 study-groups (F0 and F1 respectively) were raised on identical diets of slime mould and algae-covered plane tree bark in plentiful supply. While we know grandparents of F2 groups (F0) were also given this diet, grandparents of F1 groups were wild and their diet and nutritional intake is unknown. It is therefore possible that maternal and grandmaternal effects, comparable to those reported for *F. candida*^56^, are contributing toward the generational differences reported in this study. A test of this hypothesis was not possible, however, unpublished results indicate a growth trajectory in the F3 generation similar to that of the F2 generation.

On initial assessment the inter-generation differences observed in this study suggest that slime mould is a sub-optimal resource for *N. muscorum* as the F2 generation mature later and at a smaller size than their F1 counterparts, which is characteristic of depreciating diet quality in *F. candida*^56^. However, two important factors must be considered that strongly support slime mould as a food source. First, we have reared *N. muscorum* to four generations on this diet and, although viability of offspring was highly variable for all generations, no obvious decline in viability was observed amongst generations. Successful rearing and maintenance would not be possible if insufficient nutrition was provided. The bark-fed control group demonstrate that, when nutritional resources are poor, little growth and no reproduction occurs. Second, the control group also show a decrease in growth rate and body-length. This second point in particular suggests that the inter-generation differences are due to the aforementioned parental effects or to unknown laboratory-effects rather than resource limitation of slime mould. Various laboratory effects have been documented in cultures of *Drosophila*^26^,^59^,^60^ and, while it is suggested that caution is needed when extrapolating from model organism studies, these effects are generally believed to have negligible impacts.

Our success in creating a laboratory culture of *N. muscorum* using a slime mould diet provides us with a methodology that can be used to culture other members of the Neanuridae family in a laboratory setting. Alternative nutritional resources, or resource combinations, may provide further gains over a slime mould diet, but have not been tested. While algae-covered bark is clearly not a suitable food source for neanurid springtails it may have value beyond that of a nutrient resource in providing shelter or an egg-laying surface. Therefore, we suggest that providing bark, or an inert alternative, alongside the slime mould diet will better replicate the natural habitat of leaf litter or decaying wood in which Collembola are found^8^. We have utilised this feeding regime to raise an additional six Neanuridae species (Table 1; Supplementary Information online), and it may also be used to rear other Collembola species with suctorial mouthparts. This new method will facilitate the future use of neanurid Collembola as model taxa, so providing further opportunities to broaden current understanding of the environmental physiology of arthropods by integrating investigations of model and non-model species.

## Additional Information

**How to cite this article**: Hoskins, J. L. *et al.* Growth and reproduction of laboratory-reared neanurid Collembola using a novel slime mould diet. *Sci. Rep.* **5**, 11957; doi: 10.1038/srep11957 (2015).

## Supplementary Material

Supplementary Information

Supplementary Video 1

## Figures and Tables

**Table 1:** Species of Neanuridae (Collembola) that have been successfully reared on a diet of slime mould and algae-covered bark using the methods recommended in this study.

Subfamily	Tribe	Genus	Species	Generation
Neanurinae	Neanurini	*Neanura*	*muscorum*	F4
Neanurinae	Paleonurini	*?Australonura*	sp.	F3
Neanurinae	Paleonurini	Unknown	sp. 1	F2
Neanurinae	Anuridini	*Anurida*	c.f. *granaria*	F2
Pseudachorutinae	Pseudochorutini	?*Pseudachorutes*	sp.	F2
Neanurinae	Lobellini	Unknown	sp. 2	F2
Neanurinae	Lobellini	Unknown	sp. 3	F2

Latest generations as of February 2015. All lines are ongoing. See Supplementary Table 1 online for sample collection data.

## References

[b1] Hoffmann A. A. Physiological climatic limits in *Drosophila*: patterns and implications. J. Exp. Biol. 213, 870–880 (2010). 20190112 10.1242/jeb.037630

[b2] Van Heerwaarden B. & Sgrò C. M. Is adaptation to climate change really constrained in niche specialists? Proc. Roy. Soc. B-Biol. Sci. 281, 1790 (2014). 10.1098/rspb.2014.0396 PMC4123696 25056620

[b3] Chown S. L. & Terblanche J. S. Physiological diversity in insects: ecological and evolutionary contexts. Adv. Insect Physiol. 33, 50–152 (2007). 10.1016/S0065-2806(06)33002-0 PMC2638997 19212462

[b4] Colinet H., Sinclair B. J., Vernon P. & Renault D. Insects in fluctuating thermal environments. Annu. Rev. Entomol. 60, 123–140 (2015). 25341105 10.1146/annurev-ento-010814-021017

[b5] Sunday J. M., Bates A. E. & Dulvy N. K. Thermal tolerance and the global redistribution of animals. Nature Clim. Change. 2, 686–690 (2012).

[b6] Araújo M. B. *et al.* Heat freezes niche evolution. Ecol. Lett. 16, 1206–1219 (2013). 23869696 10.1111/ele.12155

[b7] Khaliq I., Hof C., Prinzinger R., Böhning-Gaese K. & Pfenninger M. Global variation in thermal tolerances and vulnerability of endotherms to climate change. Proc. Roy. Soc. B-Biol. Sci. 281, 1789 (2014). 10.1098/rspb.2014.1097 PMC4100521 25009066

[b8] Hopkin S. P. Biology of the Springtails (Insecta: Collembola) (Oxford University Press, 1997).

[b9] Petersen H. & Luxton M. A comparative analysis of soil fauna populations and their role in decomposition processes. Oikos. 39, 288–388 (1982).

[b10] Rusek J. Biodiversity of Collembola and their functional role in the ecosystem. Biodivers. Conserv. 7, 1207–1219 (1998).

[b11] Deharveng L. Recent advances in Collembola systematics. Pedobiologia. 48, 415–433 (2004).

[b12] Birkemoe T. & Leinaas H. P. Effects of temperature on the development of an arctic Collembola (*Hypogastrura tullbergi*). Funct. Ecol. 14, 693–700 (2000).

[b13] Birkemoe T. & Leinaas H. P. Growth and development in a high arctic Collembola: adaptive variation in local populations living in contrasting thermal environments. Ecol. Entomol. 26, 100–105 (2001).

[b14] Fountain M. T. & Hopkin S. P. *Folsomia candida* (Collembola): A ‘standard’ soil arthropod. Annu. Rev. Entomol. 50, 201–222 (2005). 15355236 10.1146/annurev.ento.50.071803.130331

[b15] Ellers J., Mariën J., Driessen G. & Van Straalen N. M. Temperature-induced gene expression associated with different thermal reaction norms for growth rate. J. Exp. Zool. Part B. 310, 137–147 (2008). 10.1002/jez.b.21194 17886827

[b16] Sørensen J. G. & Holmstrup M. Cryoprotective dehydration is widespread in Arctic springtails. J. Insect. Physiol. 57, 1147–1153 (2011). 21396373 10.1016/j.jinsphys.2011.03.001

[b17] Coulson S. *et al.* Simulated climate change: the interaction between vegetation type and microhabitat temperatures at Ny Ålesund, Svalbard. Polar Biol. 13, 67–70 (1993).

[b18] Coulson S. J., Coulson J., Leinaas H. P., Ims R. A. & Søvik G. Experimental manipulation of the winter surface ice layer: the effects on a High Arctic soil microarthropod community. Ecography. 23, 299–306 (2000).

[b19] Fountain M. T. & Hopkin S. P. A comparative study of the effects of metal contamination on Collembola in the field and in the laboratory. Ecotoxicology. 13, 573–587 (2004). 15526862 10.1023/b:ectx.0000037194.66321.2c

[b20] Block W. & Convey P. Seasonal and long-term variation in body-water content of an Antarctic springtail–a response to climate change? Polar Biol. 24, 764–770 (2001).

[b21] Slabber S., Worland M. R., Leinaas H. P. & Chown S. L. Acclimation effects on thermal tolerances of springtails from sub-Antarctic Marion Island: indigenous and invasive species. J. Insect. Physiol. 53, 113–125 (2007). 17222862 10.1016/j.jinsphys.2006.10.010

[b22] Hawes T. C., Bale J. S., Worland M. R. & Convey P. Trade-offs between microhabitat selection and physiological plasticity in the Antarctic springtail, *Cryptopygus antarcticus* (Willem). Polar Biol. 31, 681–689 (2008).

[b23] Teets N. & Denlinger D. L. Surviving in a frozen desert: environmental stress physiology of terrestrial Antarctic arthropods. J. Exp. Biol. 217, 84–93 (2014). 24353207 10.1242/jeb.089490

[b24] Van Dooremalen C., Berg M. P. & Ellers J. Acclimation responses to temperature vary with vertical stratification: implications for vulnerability of soil-dwelling species to extreme temperature events. Glob. Change Biol. 19, 975–984 (2013). 10.1111/gcb.12081 23504852

[b25] Cacoyianni Z., Kovacs I. V. & Hoffmann A. A. Laboratory adaptation and inbreeding in *Helicoverpa punctigera* (Lepidoptera: Noctuidae). Aust. J. Zool. 43, 83–90 (1995).

[b26] Sgrò C. M. & Partridge L. Evolutionary responses of the life history of wild caught *Drosophila melanogaster* to two standard methods of laboratory culture. Am. Nat. 156, 341–353 (2000).

[b27] Janion C., Leinaas H. P., Terblanche J. S. & Chown S. L. Trait means and reaction norms: the consequences of climate change/invasion interactions at the organism level. Evol. Ecol. 24, 1365–1380 (2010).

[b28] Macnamara C. The food of Collembola. Can. Entomol. 56, 99–105 (1924).

[b29] Anderson J. & Healey I. Seasonal and inter-specific variation in major components of the gut contents of some woodland Collembola. J. Anim. Ecol. 41, 359–368 (1972).

[b30] Saur É. & Ponge J.-F. Alimentary studies on the Collembolan *Paratullbergia callipygos* using transmission electron microscopy. Pedobiologia. 31, 355–379 (1988).

[b31] Janssens F. & Christiansen K. A. Class Collembola Lubbock, 1870. Animal biodiversity: An outline of higher-level classification and survey of taxonomic richness. Zootaxa. 3148, 192–194 (2011).

[b32] Cassagnau P. Sur un essai de classification des Neanuridae holarctiques et sur quelques especes de ce groupe. Rev. Fr. Entomol. 22, 134–163 (1955).

[b33] Cassagnau P. L’évolution des pieces buccales et la polytenie chez les collemboles. C. R. Acad. Sci. Paris Ser. D. 267, 106–109 (1968).

[b34] Ing B. Myxomycetes as food for other organisms. Proc. S. London Ent. Nat. Hist. Soc. 1967, 18–23 (1967).

[b35] Chassain M. Capture d’un insecte collembole par deux myxomycetes. Doc. Mycol. 2, 37–38 (1973).

[b36] Greenslade P., Moore S. & Farrow R. Observations on the feeding behaviour of Uchidanurinae (Collembola: Neanuridae) in Australia. Victorian Nat. 119, 221–223 (2002).

[b37] Smolis A. Redescription and lectotype designation of *Thaumanura carolii* (Stach, 1920) (Collembola, Neanuridae), with remarks on its biology. Deut. Entomol. Z. 56, 73–83 (2009).

[b38] Wheeler Q. D. & Miller K. B. Slime-mold beetles of the genus *Agathidium* Panzer in North and Central America, Part I. Coleoptera: Leiodidae. Bull. Am. Mus. Nat. Hist. 290, 1–95 (2005).

[b39] Southwood T. R. E. & Henderson P. A. Ecological Methods *3rd Edition*. 462–502. Wiley-Blackwell, 2000).

[b40] Janion C., Worland. M. R. & Chown S. L. Assemblage level variation in springtail lower lethal temperature: the role of invasive species on sub-Antarctic Marion Island. Physiol. Entomol. 34, 284–291 (2009).

[b41] Huber P. J. Robust Statistics (Wiley, 1981).

[b42] R Development Core Team. R: A Language and Environment for Statistical Computing (R Foundation for Statistical Computing, 2014).

[b43] Blanton R. L. Slime Moulds (Wiley, 2001).

[b44] Ruess L., Häggblom M. M., Langel R. & Scheu S. Nitrogen isotope ratios and fatty acid composition as indicators of animal diets in below ground systems. Oecologia. 139, 336–346 (2004). 15007726 10.1007/s00442-004-1514-6

[b45] Chahartaghi M., Langel R., Scheu S. & Ruess L. Feeding guilds in Collembola based on nitrogen stable isotope ratios. Soil Biol. Biochem. 37, 1718–1725 (2005).

[b46] Greenslade P., Simpson J. A. & Grgurinovic C. A. Collembola associated with fungal fruit-bodies in Australia: Proceedings of the Xth international Colloquium on Apterygota, České Budějovice 2000: Apterygota at the Beginning of the Third Millennium. Pedobiologia. 46, 345–352 (2002).

[b47] Berg M. P., Stoffer M. & van den Heuvel H. H. Feeding guilds in Collembola based on digestive enzymes. Pedobiologia. 48, 589–601 (2004).

[b48] Castaño-Meneses G., Palacios-Vargas J. G. & Cutz-Pool L. Q. Feeding habits of Collembola and their ecological niche. An. Inst. Biol. Univ. Nac. Auton. Mex. Ser. Zool. 75, 135–142 (2004).

[b49] Richards O. & Davies R. Imms’ General Textbook of Entomology. (Springer, 1977).

[b50] Deharveng L. Morphologie évolutive des Collemboles Neanurinae en particulier de la lignée Neanurienne. Travaux du Laboratoire d’Ecobiologie des Arthropodes Edaphiques, Toulouse. 4, 1–63 (1983).

[b51] Singh S. Preliminary observations on the food preference of certain Collembola (Insecta). Rev. Ecol. Biol. Sol. 6, 461–467 (1969).

[b52] Bellinger P. F., Christiansen K. A. & Janssens F. *Checklist of the Collembola of the World* (2015). Available at: www.collembola.org. (Accessed: 20th March 2015).

[b53] Cassagnau P. Parthenogenese geographique et polyploidie chez *Neanura muscorum*. C. R. Acad. Sc. Paris 274, 1846–1848 (1972).

[b54] Chernova N. M., Potapov M. B., Savenkova Y. Y. & Bokova A. I. Ecological significance of parthenogenesis in collembola. Entomol. Rev. 90, 23–38 (2010).

[b55] Tully T. & Ferrière R. Reproductive flexibility: genetic variation, genetic costs and long-term evolution in a Collembola. PLoS one 3, e3207 (2008). 18791644 10.1371/journal.pone.0003207 PMC2527682

[b56] Hafer N., Ebil S., Uller T. & Pike N. Transgenerational effects of food availability on age at maturity and reproductive output in an asexual collembolan species. Biol. Lett. 7, 755–758 (2011). 21411448 10.1098/rsbl.2011.0139 PMC3169046

[b57] Valtonen T. M., Kangassalo K., Pölkki M. & Rantala M. J. Transgenerational effects of parental larval diet on offspring development time, adult body size and pathogen resistance in *Drosophila melanogaster*. PLoS one. 7, e31611 (2012). 22359607 10.1371/journal.pone.0031611 PMC3281084

[b58] Buescher J. L. *et al.* Evidence for transgenerational metabolic programming in *Drosophila*. Dis. Model. Mech. 6, 1123–1132 (2013). 23649823 10.1242/dmm.011924 PMC3759332

[b59] Hoffmann A. A., Hallas R., Sinclair C. & Partridge L. Rapid loss of stress resistance in *Drosophila melanogaster* under adaptation to laboratory culture. Evolution. 55, 436–438 (2001). 11308098 10.1111/j.0014-3820.2001.tb01305.x

[b60] Griffiths J. A., Schiffer M. & Hoffmann A. A. Clinal variation and laboratory adaptation in the rainforest species *Drosophila birchii* for stress resistance, wing size, wing shape and development time. J. Evolution. Biol. 18, 213–222 (2005). 10.1111/j.1420-9101.2004.00782.x 15669978

