# Supplementary material for: Growth and reproduction of laboratory-reared neanurid Collembola using a novel slime mould diet
Source: Sci Rep. 2015 Jul 8;5:11957. doi: 10.1038/srep11957 (PMC4495557; doi:10.1038/srep11957)
Supplement: Supplementary Information [file srep11957-s1.pdf]

Growth and reproduction of laboratory-reared neanurid Collembola using a novel slime mould diet

Jessica L. Hoskins, Charlene Janion-Scheepers, Steven L. Chown, and Grant A. Duffy*

Supplementary Information

Rearing methods for additional species listed in Table 1

In addition to *N. muscorum*, we collected other neanurid Collembola species (Table S1) from wet eucalypt forest sites in Victoria, New South Wales, and Queensland, Australia. The leaf litter extraction methods described in the main text were used to extract Collembola and create initial stocks. For each species, initial stock sizes were typically between 20 and 50 individuals. These species were given a combined diet of slime mould and bark from *Platanus* sp. trees. All populations were maintained in temperature-controlled rooms set at 15 °C on a 12:12 hour light:dark photoperiod. Populations were regularly checked for eggs and the development time of eggs and number of generations was monitored.

Supplementary Video 1: Montage of videos and photographs of a selection of neanurid species successfully reared in the laboratory using a combined bark and slime mould diet.

Table S1: Taxonomic and sample collection data for Neanuridae species that have been successfully reared on a diet of slime mould and algae-covered bark using the methods recommended in Hoskins et al.. Latest generations (Gen.) as of February 2015. All lines are ongoing.

**Subfamily**	**Tribe**	**Genus**	**Species**	**Gen.**	**Locale**	**Lat. (°)**	**Lon. (°)**	**Elevation (m)**	**Collection date**
Neanurinae	Neanurini	*Neanura*	*muscorum*	F4	Monash University, Clayton, VIC	-37.9119	145.1317	101	10-Oct-13
Neanurinae	Paleonurini	*?Australonura*	sp.	F3	Mission Beach, QLD	-17.8591	146.0619	67	17-Jul-14
Neanurinae	Paleonurini	Unknown	sp. 1	F2	Conway National Park, QLD	-20.2872	148.7640	19	19-Jul-14
Pseudachorutinae	-	*Anurida*	c.f. *granaria*	F2	Jock Marshall Reserve, Clayton, VIC	-37.9103	145.1406	83	22-Jan-14
Pseudachorutinae	-	?*Pseudachorutes*	sp.	F2	Eungella National Park, QLD	-21.1683	148.5069	719	04-Jun-14
Neanurinae	Lobellini	Unknown	sp. 2	F2	Cotton-Bimbang National Park, NSW	-31.4937	152.6596	64	04-Jun-14
Neanurinae	Lobellini	Unknown	sp. 3	F2	Wooroonoonan National Parks, QLD	-17.6700	145.7280	602	14-Jul-14
